# Alcoholic Insanity

**Published:** 1886-01

**Authors:** 


### Editorial.

### Alcoholic Insanity.

The subject of alcoholic insanity and alcoholic trance state is certainly a most interesting one, and, at the present time, is attracting the attention of both the medical and legal professions. Within the last two years, this theory of the French writers has been advocated in this country, and it is within a shorter period that it has been received with any favor in England. It is, we may add, still in the balance to be further weighed, and it is not as yet accepted by the majority of the advanced medical thinkers, although it has strong and intelligent advocates. But what is this insanity, so-called, and why is it not accepted by all as true insanity? 1st, What do these writers call alcoholic insanity?

A prominent writer on this subject says: "I have merely to describe and illustrate those forms of mental disease in which alcohol has not only been the cause, but has so influenced the symptoms that they are in some way special or peculiar, so that the mental and bodily results are, as it were, specific, and so may be called alcoholic insanity."

"The most typical alcoholic insanity is delirium tremens, or acute alcoholism."

"That it is described in ordinary text-books on Practice and Physic, and is treated usually at home or in general hospitals, and is of short duration, does not make it less a true insanity. From a symptomological point of view, it is a typical, excited or motor melancholia, characterized especially by hallucinations of sight, fleeting delusions of all kinds, but especially delusions of suspicion, suicidal feelings, partial or complete incoherence, failure of memory, great confusion, tendency to mistake identities, in some cases by unconsciousness and by loss of power of attention."

In addition, are described bodily symptoms which are well known to us all as belonging to delirium tremens. Mania à potu is also described as acute alcoholic insanity. "The next form of alcoholic insanity is that condition commonly known as chronic alcoholism. This is also always accompanied by motor signs, many cases, indeed, not being technically 'insane.' It is often ushered in by alcoholic convulsions. In chronic alcoholism, looked at, as I am doing, chiefly from a mental point of view, all the symptoms are less acute, and last longer, than those of acute alcoholic insanity. The suspicions and fears of the latter become a chronic symptom, the delusions are less numerous, and more apt to become fixed. The hallucinations of sight are absent, but we are far more apt to have hallucinations of hearing. There is loss of inhibitory power, and, therefore, tendencies to impulsive acts." There are certain delusions which are said to belong to this chronic form, such as marital infidelity, delusions in regard to sexual relations and to poisoning. It is further maintained, that a person suffering from either of these forms of insanity, is wholly unable to judge between right and wrong. And that, associated with the chronic form, we may have a trance state in which a man may perform all of his accustomed and ordinary duties without even being suspected of being intoxicated or in an abnormal condition. This is akin to the epileptic trance state, which is recognized as a true and well-defined condition. Thus we have described, very briefly, to be sure, and imperfectly, the forms of alcoholic insanity.

Now, what do we mean by insanity? This is a hard thing to define; but for practical purposes it is said to be "a more or less prolonged departure from a man's normal mode of feeling, thinking or acting, the result of disease of the brain." This definition, as well as most of those given in the standard authors, excludes all temporary delirium, from whatsoever cause it may arise, whether from fever, from opium, or alcohol. Thus it would be seen that a temporary delirium could not be called acute insanity. Then, as to the chronic form, there are certain physical changes and certain mental symptoms which are quite persistent when the patient is intoxicated; but most of these clear up when the drink is withheld for a time, and the so-called insanity disappears. But, at times, some of these conditions are followed by insanity, and are so associated with the drinking as to appear to be due entirely to continued imbibing; the case has, however, already assumed the form of true insanity, and, if the patient be restrained from the use of alcohol, the mental symptoms and delusions are still present. That alcohol can cause brain disease, and hence can cause insanity, is a well-known fact. But to call temporary delirium from alcohol or intoxication insanity, is not correct, and is a dangerous as well as an unwarrantable position to take. While we admit that alcohol produces " a condition where the higher brain functions are suspended," and while in this condition the persons may not appreciate the difference between right and wrong and may have no recollection of anything that transpires while in this state, we cannot call it insanity, but a voluntary state induced by alcohol. A crime committed by a man suffering simply from this voluntary state of intoxication, cannot be excused and freed on the ground of insanity by medical men; but the question arising is a legal one, and the man should be held responsible. As to the delusions of the so-called alcoholic insanity, it is claimed they are pathognomonic of this form. This is another error we should not commit; for they are found in chronic mania and other forms of insanity.

We must demand, then, in view of all these facts, that we have further evidence that these states or conditions are true insanity, before we are able to accept them. The so-called trance state, due to alcohol, we will discuss at a future date.

---

So far, the medical appointments which have been made at the County Hospital and the County Insane Asylum are such as the profession can endorse most heartily. Dr. John H. Pryor, formerly resident surgeon at the Buffalo General Hospital, and, during the last few years, district physician of the sixth district, has been appointed consulting physician and surgeon to the hospital and asylum. Although quite young, Dr. Pryor will, we have no doubt, fill this position in a satisfactory manner. Dr. W. H. Slacer, who has held this position for six years, retires with the friendship of those in immediate charge, and with the satisfaction of having done his whole duty and won an enviable reputation among those who have become familiar with his work while there.

Dr. Ring is to be superintendent of the asylum as before. He was indorsed by the whole profession, and has won his place by attention to duty and a careful oversight of the institution. We wish the doctor success during the coming term of service. We sincerely hope that the resident physician to the hospital who is selected will be a man who is fitted to fill the place as well as the present incumbent. Although not a medical man, the assistant superintendent of the asylum occupies an important position, and should be a man of ability and good reputation. This will be demanded by the medical profession especially in this institution, in which we all take an interest. We can only add that the present incumbent has proved a faithful officer, and no political move should keep him from re-appointment.

---

The vital statistics for western New York show that the year 1885 has been unusually healthy. There has been a great reduction in deaths from scarlet fever, diarrhœal diseases, typhoid fever and zymotic diseases, as a class, with the exception of diphtheria, which has been more fatal than in 1884. The death rate in Buffalo for 1885 was 19 per 1,000, a reduction of 1½ per 1,000, or 7½ per cent. from the preceding year. The causes for this reduction are, first, the cholera excitement, which caused both the health authorities and the people generally to take extra precautions for the preservation of the public health. Never before in the histories of Buffalo and Rochester has there been such a war against filth, and, as a result, there has been a marked reduction in the deaths from diarrhœal diseases. Water mains have been extended; in Buffalo, twenty-nine miles of pipe were laid; hundreds of wells have been filled, and, as a consequence, the mortality from typhoid fever has been reduced fifty per cent. The second cause for the reduction of the death rate is, that the weather during 1885 has been exceptionally favorable for health. The cool summer was attended with but little fatal diarrhœa, the frequent rains kept the wells filled, the streets clean, and removed the impurities from the air, thus preventing typhoid fever and dysentery in districts where well water is used. But "it's a good wind that blows no ill," if we may be allowed to reverse the old proverb, and the same atmospheric causes which have reduced sickness from bowel diseases, have, by keeping the soil cold and moist, increased the death rate from diphtheria.

---

The Medical Society of the State of New York will hold its eightieth annual meeting in Albany, February 2, 3 and 4, 1886. The programme which we have received shows us that it will be perhaps the best meeting since this troublesome code question was introduced into the society. The committee assures us that the code question is now a thing of the past, and old and new coders alike will meet this year to carry on scientific work as before. Among the prominent physicians from the State who will be present and will read, are: Drs. A. Jacobi, V. P. Gibney, D. B. St. John Roosa, Wesley M. Carpenter, F. N. Otis, Alfred L. Loomis, W. H. Tompson, T. R. Pooley, R. F. Weir, D. H. Goodwillie, C. R. Agnew, and others, from New York; Dr. W. C. Wey and Dr. Squire, of Elmira; Dr. Ely, Dr. J. O. Roe, ot Rochester; many from Albany; Dr. Ford, of Utica, and Dr. Jewett, of Canandaigua. Buffalo will be well represented. Dr. Howe will read on the effect of the electric light on the eye; Dr. W. W. Potter, on obstetric palpation; Dr. H. R. Hopkins, on ulcerative endocarditis; Dr. Floyd S. Crego, on Migraine; Dr. C. Cary, on cremation; Dr. F. W. Hinkel, on tubercular ulceration of the pharynx. The themes of the papers of the other members are on interesting subjects, and we predict a pleasant and profitable time for those who attend.

